# Supplementary material for: A prospective population-based multicentre study on the impact of maternal body mass index on adverse pregnancy outcomes: Focus on normal weight
Source: PLoS One. 2021 Sep 23;16(9):e0257722. doi: 10.1371/journal.pone.0257722 (PMC8460045; doi:10.1371/journal.pone.0257722)

S1 Fig. Directed Acyclic Graph of the relation between body mass index (exposure) and adverse pregnancy outcome (outcome).

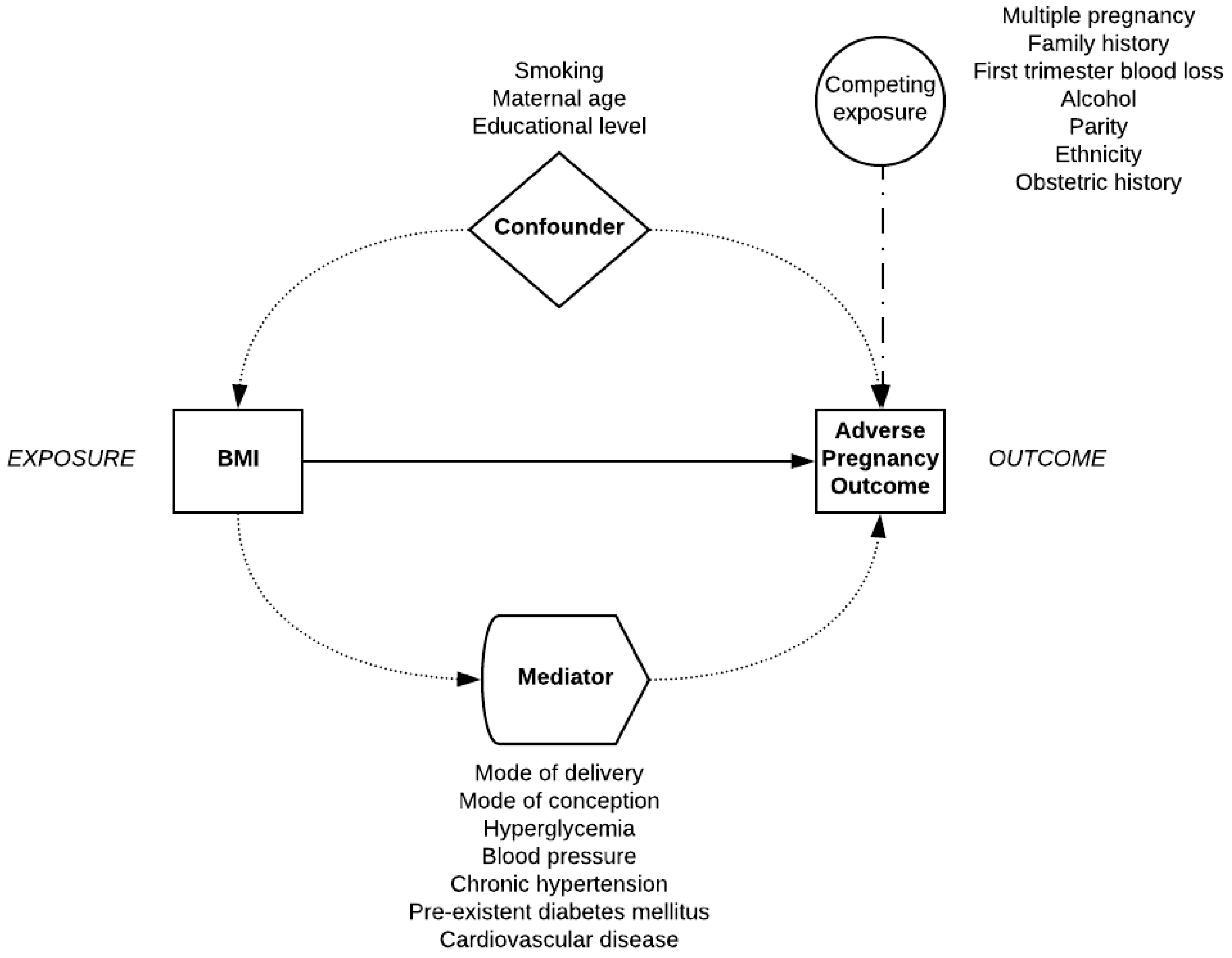

Supplement: S1 Fig — (PDF) [file pone.0257722.s001.pdf]
