# Supplementary material for: A prospective population-based multicentre study on the impact of maternal body mass index on adverse pregnancy outcomes: Focus on normal weight
Source: PLoS One. 2021 Sep 23;16(9):e0257722. doi: 10.1371/journal.pone.0257722 (PMC8460045; doi:10.1371/journal.pone.0257722)
Supplement: S1 Table — (PDF) [file pone.0257722.s002.pdf]

S1 Table. Baseline characteristics of patients in the RESPECT cohort, stratified by variables that were available for imputation.

Characteristic	Patients with missing information	Complete cases (n=2603)	Patients with at least one missing value (n=1120)	P	Overall RESPECT cohort (n=3723)*
Age (years)	168 (4.5)	30.9 (4.34)†	30.7 (3.91)†	0.29	30.8 (4.2)†
Body mass index, before pregnancy	46 (1.2)	23.3 (21.2-26.3)†	23.0 (20.9-25.9)†	0.01‡	23.2 (21.1-26.2)†
Body mass index	182 (4.8)	23.8 (21.6-26.9)†	23.4 (21.3-26.3)†	0.01‡	23.7 (21.5-26.7)†
Systolic blood pressure (mm Hg)	65 (1.7)	114 (12)†	115 (12)†	0.48	115 (12)†
Diastolic blood pressure (mm Hg)	64 (1.7)	67 (8)†	67 (8)†	0.09	67 (8)†
Glucose (mmol/L)	171 (4.5)	4.7 (4.3-5.1)†	4.7 (4.4-5.1)†	0.34	4.7 (4.4-5.1)†
Ethnicity					
White		1665 (89.0)	1066 (95.2)		3387 (91.0)
African		17 (0.9)	2 (0.2)		30 (0.8)
Asian	732 (19.7)	30 (1.6)	11 (1.0)	<0.001‡	53 (1.4)
Mixed		44 (2.4)	15 (1.3)		77 (2.1)
Other		115 (4.4)	26 (2.3)		176 (4.7)
Education					
Low		198 (7.6)	52 (4.6)		270 (7.3)
Middle	223 (6.0)	825 (31.7)	362 (32.3)	0.004‡	1273 (34.29)
High		1357 (57.0)	706 (63.0)		2180 (58.6)
Smoking during pregnancy	0	258 (9.9)	73 (6.5)	0.001‡	334 (9.0)
History of chronic hypertension	1 (0.0)	43 (1.7)	14 (1.2)	0.44	57 (1.5)
Family history of diabetes mellitus	1 (0.0)	389 (15.0)	154 (13.8)	0.37	543 (14.6)
Method of conception					
Spontaneous		2396 (93.1)	1033 (92.2)		3429 (92.9)
Ovulation drugs	30 (0.8)	61 (2.4)	38 (3.4)	0.20	99 (2.7)
In vitro fertilisation		82 (3.2)	28 (2.5)		110 (3.0)
Nulliparous	4 (0.0)	1143 (44.0)	509 (45.4)	0.43	1655 (44.5)
History of gestational diabetes mellitus	0	47 (1.8)	12 (1.1)	0.13	59 (1.6)
History of macrosomia (>90th percentile)	0	146 (5.6)	84 (7.5)	0.03‡	230 (6.2)
Recurrent miscarriages (≥2)	4 (0.0)	173 (6.7)	59 (5.3)	0.13	232 (6.2)
History of fetal death	0	58 (2.2)	16 (1.4)	0.14	74 (2.0)
Gestational diabetes mellitus in pregnancy	263 (7.0)	116 (5.0)	53 (4.7)	0.84	181 (4.9)
Insulin dependent		20 (0.8)	13 (1.2)	0.33	33 (0.9)
Gestational age at delivery (days)	342 (9.2)	280 (273-285)†	280 (274-286)†	0.36	280 (273-285)†
Sex (male)	358 (9.6)	1154 (50.7)	569 (52.3)	0.40	1902 (51.1)
Birthweight (g)		3504 (3200-3860)†	3540 (3216-3880)†	0.13	3520 (3190-3880)†
Percentile	372 (10.0)	55 (30-77)†	57 (32-80)†	0.07	55 (30-79)†
>90th percentile		256 (12.0)	140 (13.2)	0.37	494 (13.3)

Data are no (%) unless stated otherwise. *Data in column includes imputed data for those with missing values. †Data are mean (standard deviation) or median (interquartile range). ‡Significant at the P<0.05 level. Adopted from the external validation study previously published by our group (Lamain-de Ruiter M et al. External validation of prognostic models to predict risk of gestational diabetes mellitus in one Dutch cohort: prospective multicentre cohort study. BMJ. 2016;354:i4338).
