# Supplementary material for: A prospective population-based multicentre study on the impact of maternal body mass index on adverse pregnancy outcomes: Focus on normal weight
Source: PLoS One. 2021 Sep 23;16(9):e0257722. doi: 10.1371/journal.pone.0257722 (PMC8460045; doi:10.1371/journal.pone.0257722)
Supplement: S1 File — (PDF) [file pone.0257722.s003.pdf]

RESPECT STUDY

Questionnaire - first visit

Terms within quotation marks (“,“) indicate words or terms from the original Dutch questionnaire without direct possible translations.

The original questionnaire was issued to participants in the Dutch language; this English version serves as supplemental information to this publication.

Dear madam,

As a participant of the RESPECT-study, we kindly ask you to fill in this questionnaire after your first prenatal visit. It is possible that questions from this questionnaire overlap with the information that you have already provided prior to or during your first visit. For this study, we ask you to answer these again. You can use the provided envelope to return your finished questionnaire.

We thank you for your cooperation.

The RESPECT-study team (respectstudie@umcutrecht.nl)

GENERAL QUESTIONS

What is today's date? __-__-____ (dd-mm-yyyy)

What is your zip code? ____-__

What is your date of birth? __-__-____ (dd-mm-yyyy)

In which country were you born?

- The Netherlands
- Belgium
- Germany
- Turkey
- Morocco
- Suriname
- Dutch Antilles
- Indonesia
- A different country, namely…………..

In which country were your (biological) mother and father born?

Own **mother**:

- The Netherlands
- Belgium
- Germany
- Turkey
- Morocco
- Suriname
- Dutch Antilles
- Indonesia
- A different country, namely…………..

Own **father**:

- The Netherlands
- Belgium
- Germany
- Turkey
- Morocco
- Suriname
- Dutch Antilles
- Indonesia

- A different country, namely………….

How would you classify your ethnic background?

- Caucasian (Dutch, white-European (not including Turkey)
- African (African, Surinamese / Antillean of black descent)
- Hindustani (Hindustani, Pakistani, Indian, Surinamese / Antillean of Hindustani descent)
- Moroccan (Moroccan, Algerian, North-African
- Turkish (Turkish, Kurdish)
- Middle-Eastern (Iranian, Iraqi, Afghani)
- Asian (Chinese, Japanese, Indonesian, Ambonese, Vietnamese)
- Other Western (North-American, Australian)
- Other non-Western (South- and Central-American)
- Mixed (mixed ethnic background)

What is your highest finished education?

- Primary school
- Secondary school
  - "LBO"
  - "VMBO"
  - "MAVO"
  - "HAVO"
  - "VWO"
  - other
- "MBO"
- "HBO"
- University

How old were you when you finished this education?

SMOKING AND ALCOHOL CONSUMPTION

Have you smoked cigarettes or rolling tobacco in the last week?

- No
- Yes, on average less than one cigarette or piece of rolling tobacco a day
- Yes, on average ….. cigarettes or pieces of rolling tobacco a day

Did you smoke prior to your current pregnancy?

- No, I have never smoked
- No, I quit smoking in ____ (calendar year)
- Yes, prior to this pregnancy I smoked on average ….. cigarettes or pieces of rolling tobacco a day

Have you consumed alcohol in the last week?

- No
- Yes, a total of ….. glasses

Did you consume alcohol prior to your current pregnancy?

- No
- Yes, prior to this pregnancy I consumed on average ….. glasses of alcohol in one week

DIET

Have you been taking any extra vitamins or dietary supplements prior to or during this pregnancy?

- o No → you can skip the next question
- o Yes

Can you indicate which vitamins or dietary supplements you have been using and during which period?

	Prior to this pregnancy	During this pregnancy
o Folic acid	____ weeks	from week ____ to week ____ **or: until now**
o Vitamin C	____ weeks	from week ____ to week ____ **or: until now**
o Vitamin D	____ weeks	from week ____ to week ____ **or: until now**
o Calcium	____ weeks	from week ____ to week ____ **or: until now**
o Multivitamins	____ weeks	from week ____ to week ____ **or: until now**

How many servings of fruit do you consume on average each day?
One portion is comparable to an apple or a handful of grapes/strawberries
_____ portions of fruit

OCCUPATION

Do you have a paid occupation?

- o No → you can skip the next question
- o Yes → on average, for _____ hours a week

Do you work night shifts?

- o No
- o Yes

YOUR HEALTH

How tall are you?
_____ centimetres

How much did you weight right before your pregnancy?
_____kilogrammes

Have you been diagnosed with any of the following conditions by a doctor or physician?
You can choose multiple answers. Conditions that have only occurred during pregnancy are excluded.

- o High blood pressure
- o Diabetes mellitus type I or type II
- o Cardiovascular disease

- An episode of thrombosis
- SLE (Systemic lupus erythematodes)
- Kidney disease
- APS (Antiphospholipid syndrome)

Do you take any medication for a high blood pressure or diabetes?
- No
- Yes, namely…………………………………………………………………………………………………….

HEALTH CONDITIONS IN YOUR FAMILY

Do any of the following conditions occur in your family?
(Please tick all that apply)

	Father	Mother	Brother(s)	Sister(s)
Diabetes mellitus	…..	…..	…..	…..
High blood pressure	…..	…..	…..	…..
Cardiovascular disease	…..	…..	…..	…..

Do any of the following conditions occur more frequently in your family than is to be expected?
(Please tick all that apply)
- No
- Yes, Diabetes mellitus type I or type II
- Yes, high blood pressure
- Yes, Cardiovascular disease

Have your mother or sister(s) (if applicable) experienced any of the following problems during pregnancy?
(Please tick all that apply)

	Mother	Sister(s)
Gestational diabetes	…..	…..
Preeclampsia	…..	…..
HELLP-syndrome	…..	…..
Preterm birth (delivery under 37 weeks of gestation)	…..	…..
Delivery of a baby with a low birth weight (less than 2500gr)	…..	…..
Delivery of a baby with a high birth weight (more than 4500gr)	…..	…..

What was your own weight at birth?
- ______grams
- I don't know exactly, but my birth weight was:
  - Normal
  - Too low
  - Too high
- I don't know

Were you born at term, premature or past the due date?
- At term
- Premature, namely ____ weeks too early
- Past the due date, namely ____ too late
- I don't know

YOUR CURRENT PREGNANCY

What was the first day of your last menstruation?
__-__-____ (dd-mm-yyyy)

How many weeks are you pregnant at this moment?
__ weeks and __ days

Is it a multiple pregnancy?
- No
- Yes, a monochorionic pregnancy (a shared placenta)
- Yes, a dichorionic pregnancy (two separate placentas)
- Yes, a triplet

How did you conceive?
- Naturally
- With the use of medication that triggers an ovulation
- Insemination, without the use of additional medication
- Insemination, with the use of additional medication
- IVF/ICSI
- Another way, namely……………………………………………

After deciding to get pregnant, how many months did it take for you to actually get pregnant?
Please fill in the number of months of unprotected intercourse; If you have experienced one or multiple miscarriages, please start counting after the last miscarriage.
_____ months
- Or: the current pregnancy was unintended

Have you experienced any vaginal blood loss in the first 3 months of the current pregnancy?
This also includes minimal, brownish/old or short lasting blood loss.
- No → you can skip the next question
- Yes

How many days was the longest consecutive period in which you experienced blood loss daily?
_____ days

Is this your first pregnancy?
- No
- Yes → you are now finished with this questionnaire

Was your previous pregnancy conceived with the same partner as the current pregnancy?
- No
- Yes

PREVIOUS PREGNANCIES

Please fill in additional information regarding your previous pregnancy/pregnancies. Please include pregnancies that resulted in a (premature)birth, as well as miscarriages. If you don't know the exact details, fill in any information you do know. Please use separate lines for multiple pregnancies.

	**First pregnancy**	**Second pregnancy**
Date of delivery / miscarriage	__-__-____	__-__-____
Duration of pregnancy	__ weeks and __ days	__ weeks and __ days
Single- or multiple pregnancy	○ Single<br>○ Multiple	○ Single<br>○ Multiple
Birth weight	____grams<br>○ I don't know	____grams<br>○ I don't know
Sex of the baby	○ Boy<br>○ Girl	○ Boy<br>○ Girl
Was the baby alive at birth?	○ Yes<br>○ No	○ Yes<br>○ No
Did any of the following problems occur during this pregnancy?	○ Preeclampsia<br>○ HELLP-syndrome<br>○ Gestational diabetes<br>○ Placental abruption	○ Preeclampsia<br>○ HELLP-syndrome<br>○ Gestational diabetes<br>○ Placental abruption
Was this problem / were these problems treated using medication?	○ Yes<br>○ No	○ Yes<br>○ No
Were you admitted to the hospital because of this problem / these problems?	○ Yes<br>○ No	○ Yes<br>○ No
Did you deliver by caesarean section?	○ Yes<br>○ No	○ Yes<br>○ No

	**Third pregnancy**	**Fourth pregnancy**
Date of delivery / miscarriage	__-__-____	__-__-____
Duration of pregnancy	__ weeks and __ days	__ weeks and __ days
Single- or multiple pregnancy	○ Single<br>○ Multiple	○ Single<br>○ Multiple
Birth weight	____grams<br>○ I don't know	____grams<br>○ I don't know
Sex of the baby	○ Boy<br>○ Girl	○ Boy<br>○ Girl
Was the baby alive at birth?	○ Yes<br>○ No	○ Yes<br>○ No
Did any of the following problems occur during this pregnancy?	○ Preeclampsia<br>○ HELLP-syndrome<br>○ Gestational diabetes<br>○ Placental abruption	○ Preeclampsia<br>○ HELLP-syndrome<br>○ Gestational diabetes<br>○ Placental abruption
Was this problem / were these problems treated using medication?	○ Yes<br>○ No	○ Yes<br>○ No
Were you admitted to the hospital because of this	○ Yes<br>○ No	○ Yes<br>○ No

problem / these problems?		
Did you deliver by caesarean section?	○ Yes<br>○ No	○ Yes<br>○ No

	**Fifth pregnancy**	**Sixth pregnancy**
Date of delivery / miscarriage	__-__-____	__-__-____
Duration of pregnancy	__ weeks and __ days	__ weeks and __ days
Single- or multiple pregnancy	○ Single<br>○ Multiple	○ Single<br>○ Multiple
Birth weight	____grams<br>○ I don't know	____grams<br>○ I don't know
Sex of the baby	○ Boy<br>○ Girl	○ Boy<br>○ Girl
Was the baby alive at birth?	○ Yes<br>○ No	○ Yes<br>○ No
Did any of the following problems occur during this pregnancy?	○ Preeclampsia<br>○ HELLP-syndrome<br>○ Gestational diabetes<br>○ Placental abruption	○ Preeclampsia<br>○ HELLP-syndrome<br>○ Gestational diabetes<br>○ Placental abruption
Was this problem / were these problems treated using medication?	○ Yes<br>○ No	○ Yes<br>○ No
Were you admitted to the hospital because of this problem / these problems?	○ Yes<br>○ No	○ Yes<br>○ No
Did you deliver by caesarean section?	○ Yes<br>○ No	○ Yes<br>○ No
