# Supplementary material for: A prospective population-based multicentre study on the impact of maternal body mass index on adverse pregnancy outcomes: Focus on normal weight
Source: PLoS One. 2021 Sep 23;16(9):e0257722. doi: 10.1371/journal.pone.0257722 (PMC8460045; doi:10.1371/journal.pone.0257722)
Supplement: S2 File — (PDF) [file pone.0257722.s004.pdf]

Studienummer **01-0001**

RESPECT

Studie

Vragenlijst eerste controle

Vragenlijst eerste controle Studienummer **01-0001**

Geachte mevrouw,

In verband met het onderzoek waar u aan meedoet (RESPECT-studie), vragen wij u om deze vragenlijst in te vullen na de eerste controle. Het is mogelijk dat vragen in deze lijst overeenkomen met vragen die u al heeft beantwoord voorafgaand of tijdens de eerste controle. Voor het onderzoek willen wij u toch vragen deze **opnieuw** te beantwoorden.
De ingevulde vragenlijst kunt u opsturen door middel van de bijgevoegde antwoordenveloppe.

Hartelijk dank voor uw medewerking,

Het RESPECT-onderzoeksteam (respectstudie@umcutrecht.nl)

Algemene vragen

Wat is de datum van vandaag? ☐☐-☐☐-☐☐☐☐ (dd-mm-jjjj)

Wat is uw postcode? ☐☐☐☐-☐☐

Wat is uw geboortedatum? ☐☐-☐☐-☐☐☐☐ (dd-mm-jjjj)

In welk land bent u geboren?

- 0 Nederland
- 0 België
- 0 Duitsland
- 0 Turkije
- 0 Marokko
- 0 Suriname
- 0 Nederlandse Antillen
- 0 Indonesië
- 0 ander land, namelijk: ………………………………

Vragenlijst eerste controle   Studienummer **01-0001**

In welk land zijn uw eigen (biologische) moeder en vader geboren?

Eigen **moeder**:	Eigen **vader**:
0 Nederland	0 Nederland
0 België	0 België
0 Duitsland	0 Duitsland
0 Turkije	0 Turkije
0 Marokko	0Marokko
0 Suriname	0 Suriname
0 Nederlandse Antillen	0 Nederlandse Antillen
0 Indonesië	0 Indonesië
0 ander land, namelijk: ……………	0 ander land, namelijk:………….………..

Tot welke etnische afkomst rekent u zich?

0 Kaukasisch	(Nederlands, blank-europees (excl Turkije))
0 Afrikaans	(Afrikaans, Surinaams/Antiliaans van negroïde afkomst)
0 Hindoestaans	(Hindoestaans, Pakistaans, Indiaas, Surinaams/Antilliaans van hindoestaanse afkomst)
0 Marokkaans	(Marokkaans, Algerijns, Noord-Afrikaans)
0 Turks	(Turks, Koerdisch)
0 Midden-Oosten	(Iranees, Irakees, Afghaan)
0 Aziatisch	(Chinees, Japans, Indonesisch, Ambonees, Vietnamees)
0 overig Westers	(Noord-Amerikaans, Australisch)
0 overig Niet-Westers	(Zuid- en Midden-Amerikaans)
0 gemengd	(gemengde afkomst)

Wat is de <u>hoogste</u> opleiding die u heeft afgemaakt?

0 lagere school / basisschool
0 middelbaar onderwijs
  0 LBO - 0 VMBO - 0 MAVO – 0 HAVO – 0 VWO – 0 anders
0 MBO
0 HBO
0 universitaire opleiding

Hoe oud was u toen u deze opleiding voltooide? ☐☐ jaar

Vragenlijst eerste controle   Studienummer **01-0001**

Roken en alcoholgebruik

Heeft u in de afgelopen week sigaretten of shagjes gerookt?

0 Nee
0 Ja, gemiddeld minder dan één sigaret of shagje per dag
0 Ja, gemiddeld ☐☐ sigaret(ten)/shagje(s) per dag

Rookte u voor uw huidige zwangerschap?

0 Nee, ik heb nooit gerookt
0 Nee, ik ben gestopt met roken in ☐☐☐☐ (kalenderjaar noemen)
0 Ja, ik rookte voordat ik zwanger werd gemiddeld ☐☐ sigaret(ten)/shagje(s) per dag

Heeft u in de afgelopen week alcohol gedronken?

0 Nee
0 Ja, in totaal ☐☐ glazen

Dronk u alcohol voor uw huidige zwangerschap?

0 Nee
0 Ja, voordat ik zwanger werd dronk ik gemiddeld ☐☐ glas/glazen per week

Voeding

Heeft u vóór of tijdens de zwangerschap extra vitaminen of andere voedingssupplementen geslikt?

0 Nee → de volgende vraag kunt u overslaan
0 Ja

Vragenlijst eerste controle Studienummer **01-0001**

Kunt u aangeven welke vitaminen / voedingssupplementen u heeft gebruikt en in welke periode?

	Vóór de zwangerschap:	Tijdens de zwangerschap:
□ Foliumzuur	☐☐ weken	vanaf week ☐☐ tot week ☐☐ ***of*** **:** tot nu □
□ Vitamine C	☐☐ weken	vanaf week ☐☐ tot week ☐☐ ***of*** **:** tot nu □
□ Vitamine D	☐☐ weken	vanaf week ☐☐ tot week ☐☐ ***of*** **:** tot nu □
□ Calciumtabletten	☐☐ weken	vanaf week ☐☐ tot week ☐☐ ***of*** **:** tot nu □
□ Multivitaminen	☐☐ weken	vanaf week ☐☐ tot week ☐☐ ***of*** **:** tot nu □

Hoeveel porties fruit eet u gemiddeld per dag?

Eén portie is vergelijkbaar met een appel of hand druiven/aardbeien.

☐☐ porties fruit

Werk

Verricht u betaald werk?

0 Nee → de volgende vraag kunt u overslaan

0 Ja → gemiddeld aantal uren per week ☐☐

Draait u nachtdiensten?

0 Nee

0 Ja

Uw gezondheid

Wat is uw lengte?

☐☐☐ centimeter

Wat was uw gewicht net voordat u zwanger werd?

☐☐☐ kilogram

Vragenlijst eerste controle Studienummer **01-0001**

Heeft een arts een van de volgende aandoeningen bij u vastgesteld?
U kunt meerdere antwoorden aankruisen. Aandoeningen die alleen tijdens een zwangerschap voorkwamen, hier niet aankruisen.

0 hoge bloeddruk
0 suikerziekte type 1/type 2
0 hart- en/of vaatziekten
0 ooit trombose gehad
0 SLE (Systemische lupus erythematodes)
0 nierziekte
0 APS (antifosfolipiden-syndroom)

Gebruikt u medicijnen voor een hoge bloeddruk of suikerziekte?

0 Nee
0 Ja, namelijk: …………………………………………………………………………

Ziekten in uw familie

Komt er in uw familie een of meer van de volgende aandoeningen voor?
(aankruisen waar van toepassing)

	Vader	Moeder	Broer(s)	Zus(sen)
Suikerziekte	□	□	□	□
Hoge bloeddruk	□	□	□	□
Hart- en/of vaatziekten	□	□	□	□

Komt een van deze aandoeningen veel meer voor dan verwacht in uw familie? (meerdere antwoorden mogelijk)

0 Nee
0 Ja, suikerziekte
0 Ja, hoge bloeddruk
0 Ja, hart- en/of vaatziekten

Vragenlijst eerste controle Studienummer **01-0001**

Hebben uw moeder of eventuele zussen de volgende problemen in de zwangerschap gehad? (aankruisen waar van toepassing)

	Moeder	Zus(sen)
Zwangerschapssuiker (zwangerschapsdiabetes)	□	□
Zwangerschapsvergiftiging (pre-eclampsie)	□	□
HELLP-syndroom	□	□
Vroeggeboorte (bevalling voor 37 weken zwangerschap)	□	□
Kind met een laag geboortegewicht (minder dan 2500 gram)	□	□
Kind met een hoog geboortegewicht (meer dan 4500 gram)	□	□

Wat was uw eigen geboortegewicht?

☐☐☐☐ gram

0 Weet ik niet precies, maar gewicht was: 0 normaal - 0 te laag - 0 te hoog
0 Weet ik niet

Bent u ‘op tijd’, ‘te vroeg’ of ‘te laat’ geboren?

0 Op tijd
0 Te vroeg, namelijk ☐☐ weken te vroeg
0 Te laat, namelijk ☐☐ weken te laat
0 Weet ik niet

Uw huidige zwangerschap

Wat is de eerste dag van uw laatste menstruatie? ☐☐-☐☐-☐☐☐☐ (dd-mm-jjjj)

Hoeveel weken bent u nu zwanger? ☐☐ weken en ☐ dagen

Is er sprake van een meerlingzwangerschap?

0 Nee
0 Ja, een monochoriale tweeling (een gedeelde placenta)
0 Ja, een bichoriale tweeling (twee aparte placenta’s)
0 Ja, een drieling

Vragenlijst eerste controle Studienummer **01-0001**

Hoe is uw huidige zwangerschap tot stand gekomen?

- 0 via de natuurlijke weg
- 0 met medicijnen om een eisprong op te wekken
- 0 inseminatie zonder medicijnen
- 0 inseminatie met medicijnen
- 0 IVF / ICSI
- 0 andere manier, namelijk ……………………………………………….

Hoeveel maanden heeft het geduurd voordat u zwanger werd, nadat u besloten heeft zwanger te willen worden?
Vul hier het aantal maanden in dat u onbeschermde gemeenschap heeft gehad; als u een of meer miskramen heeft gehad begint u na de laatste miskraam te tellen.

☐☐ maanden
of □ het was niet de bedoeling om zwanger te worden

Heeft u in de eerste 3 maanden van huidige zwangerschap last gehad van bloedverlies uit de vagina?
Weinig, bruinig of kortdurend bloedverlies a.u.b. ook meetellen.

- 0 Nee, geen bloedverlies → ga naar vraag …
- 0 Ja

Hoe lang duurde de langst aaneengesloten periode waarin u elke dag bloedverlies had?

☐☐ dagen

Bent u voor de eerste keer zwanger?

- 0 Nee
- 0 Ja → u bent nu klaar met het invullen van de vragenlijst

Werd uw vorige zwangerschap verwekt door dezelfde partner als de huidige zwangerschap?

- 0 Nee
- 0 Ja

Vragenlijst eerste controle Studienummer **01-0001**

Eerdere zwangerschappen

Zou u hieronder een aatal gegevens willen invullen over uw eerdere zwangerschap(pen)? Het gaat hier zowel om zwangerschappen die tot een (vroeg-)*geboorte* hebben geleid als om eventuele *miskramen*.

Indien u de precieze gegevens niet weet, vult u dan de gegevens in die u wel weet.

Vult u voor meerlingen alstublieft voor elk kind een aparte rij in.

	1e zwangerschap	2e zwangerschap
Datum bevalling/miskraam	☐☐/☐☐/☐☐☐☐	☐☐/☐☐/☐☐☐☐
Duur van de zwangerschap	☐☐ weken en<br>☐☐ dagen	☐☐ weken en<br>☐☐ dagen
Eenling- of meerlingzwangerschap	□ eenling<br>□ meerling	□ eenling<br>□ meerling
Gewicht van het kind	☐☐☐☐ gram<br>Weet ik niet □	☐☐☐☐ gram<br>Weet ik niet □
Geslacht van het kind	□ jongen<br>□ meisje	□ jongen<br>□ meisje
Werd het kind leven geboren?	□ ja<br>□ nee	□ ja<br>□ nee
Was er sprake van een van de volgende problemen tijdens de zwangerschap?	□ preeclampsie<br>□ HELLP -syndroom<br>□ zwangerschapssuiker (diabetes)<br>□ loslatende placenta (moederkoek)	□ preeclampsie<br>□ HELLP -syndroom<br>□ zwangerschapssuiker (diabetes)<br>□ loslatende placenta (moederkoek)
Is dit proble(e)m(en) behandeld met medicijnen?	□ ja<br>□ nee	□ ja<br>□ nee
Bent u hiervoor opgenomen in het ziekenhuis?	□ ja<br>□ nee	□ ja<br>□ nee
Bent u bevallen via een keizersnee?	□ ja<br>□ nee	□ ja<br>□ nee

Vragenlijst eerste controle Studienummer **01-0001**

	3e zwangerschap	4e zwangerschap
Datum bevalling/miskraam	□□/□□/□□□□	□□/□□/□□□□
Duur van de zwangerschap	□□ weken en<br>□□ dagen	□□ weken en<br>□□ dagen
Eenling- of meerlingzwangerschap	□ eenling<br>□ meerling	□ eenling<br>□ meerling
Gewicht van het kind	□□□□ gram<br>Weet ik niet □	□□□□ gram<br>Weet ik niet □
Geslacht van het kind	□ jongen<br>□ meisje	□ jongen<br>□ meisje
Werd het kind leven geboren?	□ ja<br>□ nee	□ ja<br>□ nee
Was er sprake van een van de volgende problemen tijdens de zwangerschap?	□ preeclampsie<br>□ HELLP -syndroom<br>□ zwangerschapssuiker (diabetes)<br>□ loslatende placenta (moederkoek)	□ preeclampsie<br>□ HELLP -syndroom<br>□ zwangerschapssuiker (diabetes)<br>□ loslatende placenta (moederkoek)
Is dit proble(e)m(en) behandeld met medicijnen?	□ ja<br>□ nee	□ ja<br>□ nee
Bent u hiervoor opgenomen in het ziekenhuis?	□ ja<br>□ nee	□ ja<br>□ nee
Bent u bevallen via een keizersnee?	□ ja<br>□ nee	□ ja<br>□ nee

Vragenlijst eerste controle Studienummer **01-0001**

	5e zwangerschap	6e zwangerschap
Datum bevalling/miskraam	□□/□□/□□□□	□□/□□/□□□□
Duur van de zwangerschap	□□ weken en<br>□□ dagen	□□ weken en<br>□□ dagen
Eenling- of meerlingzwangerschap	□ eenling<br>□ meerling	□ eenling<br>□ meerling
Gewicht van het kind	□□□□ gram<br>Weet ik niet □	□□□□ gram<br>Weet ik niet □
Geslacht van het kind	□ jongen<br>□ meisje	□ jongen<br>□ meisje
Werd het kind leven geboren?	□ ja<br>□ nee	□ ja<br>□ nee
Was er sprake van een van de volgende problemen tijdens de zwangerschap?	□ preeclampsie<br>□ HELLP -syndroom<br>□ zwangerschapssuiker (diabetes)<br>□ loslatende placenta (moederkoek)	□ preeclampsie<br>□ HELLP -syndroom<br>□ zwangerschapssuiker (diabetes)<br>□ loslatende placenta (moederkoek)
Is dit proble(e)m(en) behandeld met medicijnen?	□ ja<br>□ nee	□ ja<br>□ nee
Bent u hiervoor opgenomen in het ziekenhuis?	□ ja<br>□ nee	□ ja<br>□ nee
Bent u bevallen via een keizersnee?	□ ja<br>□ nee	□ ja<br>□ nee
