# Supplementary material for: A prospective population-based multicentre study on the impact of maternal body mass index on adverse pregnancy outcomes: Focus on normal weight
Source: PLoS One. 2021 Sep 23;16(9):e0257722. doi: 10.1371/journal.pone.0257722 (PMC8460045; doi:10.1371/journal.pone.0257722)
Supplement: S3 File — (PDF) [file pone.0257722.s005.pdf]

S3 File. Correlation between prepregnancy and first-trimester body mass index.

Plots and correlations are displayed for the original dataset and each of the imputated datasets.

Original dataset

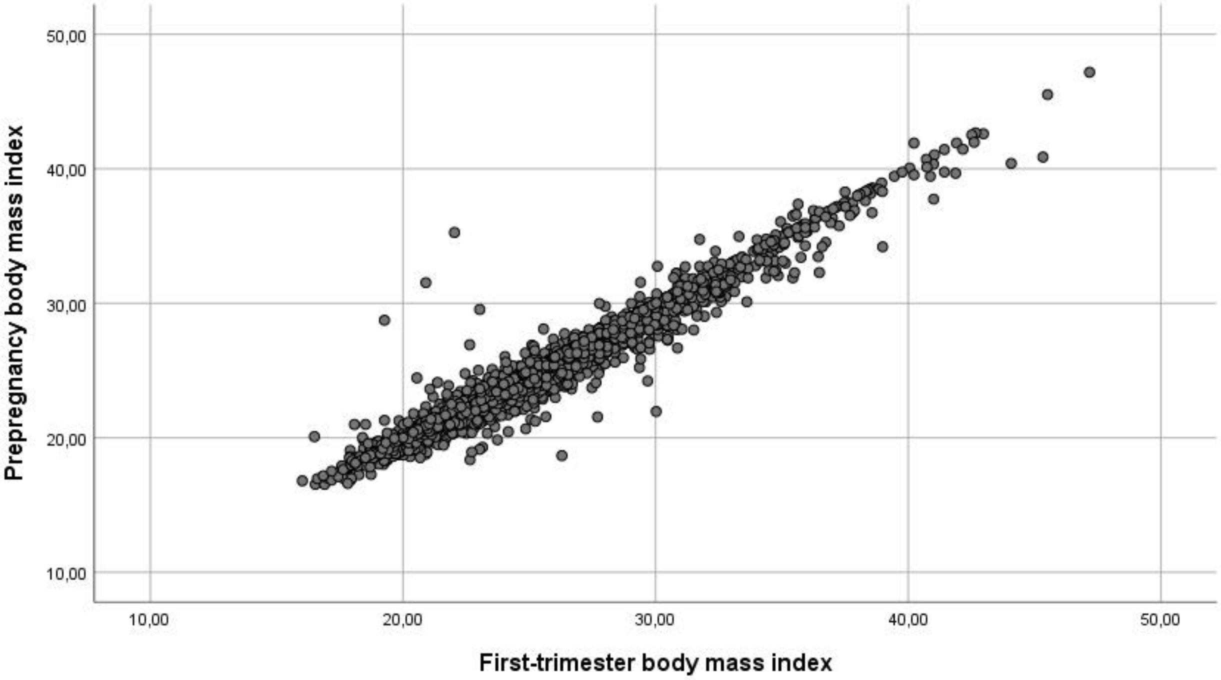

r 0.978

p-value <0.001

Imputation 1

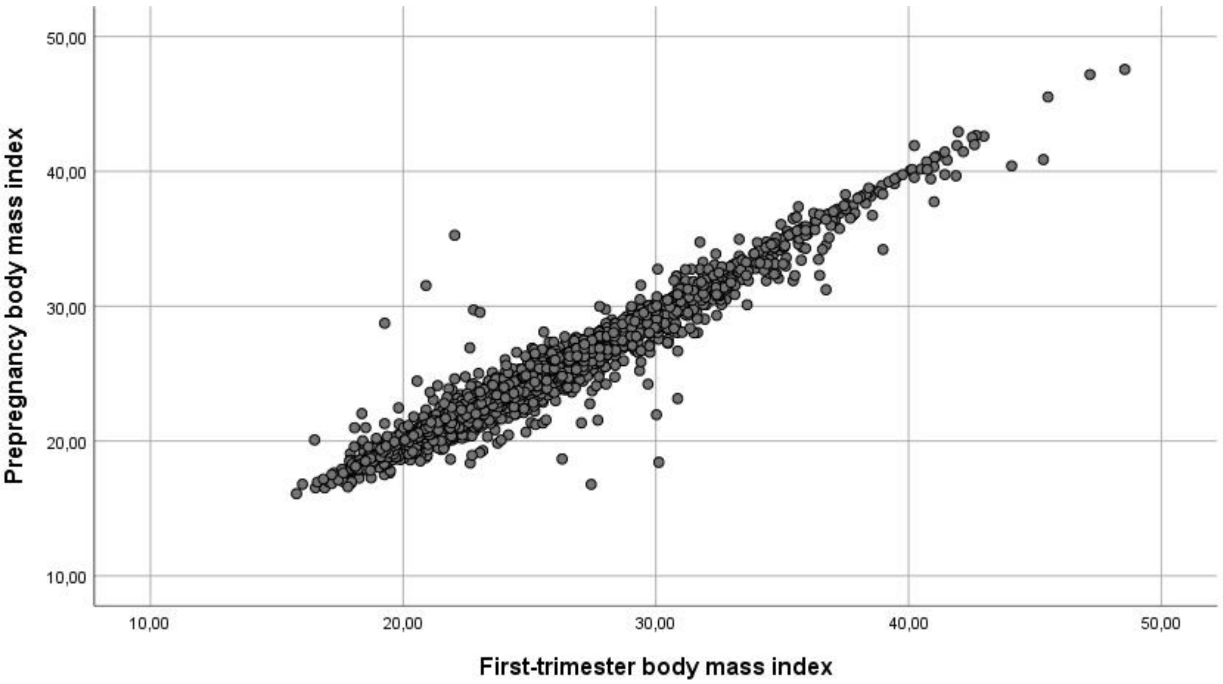

r 0.976

p-value <0.001

Imputation 2

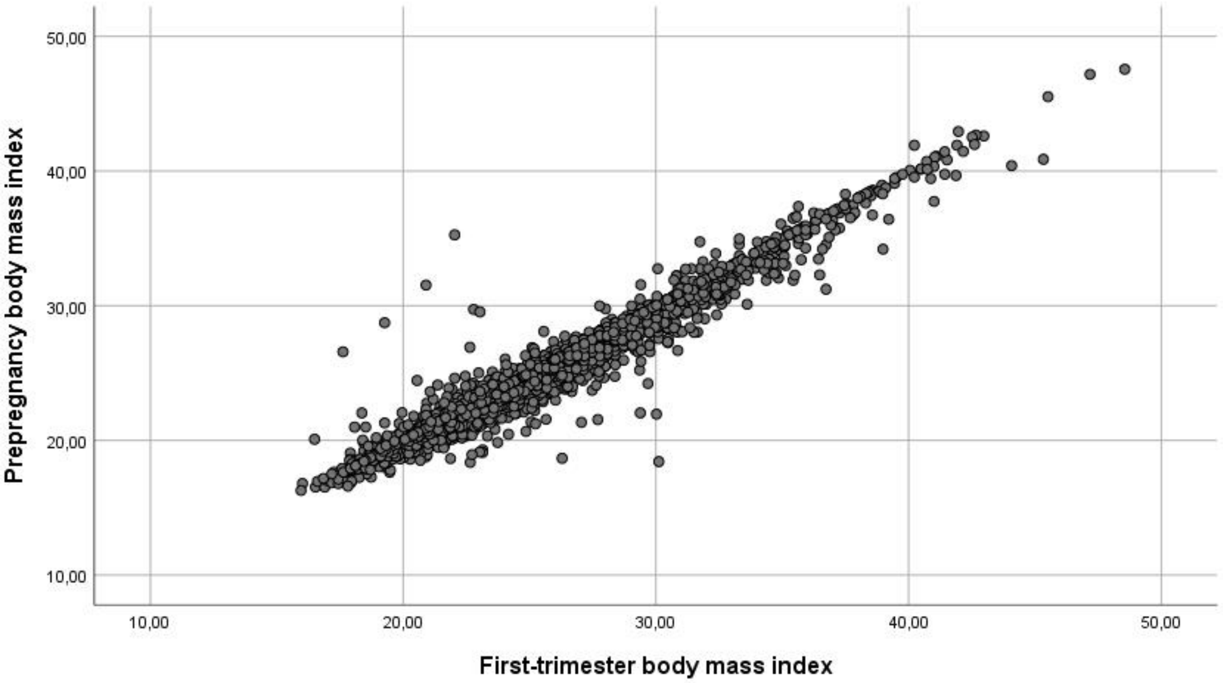

r 0.977

p-value <0.001

Imputation 3

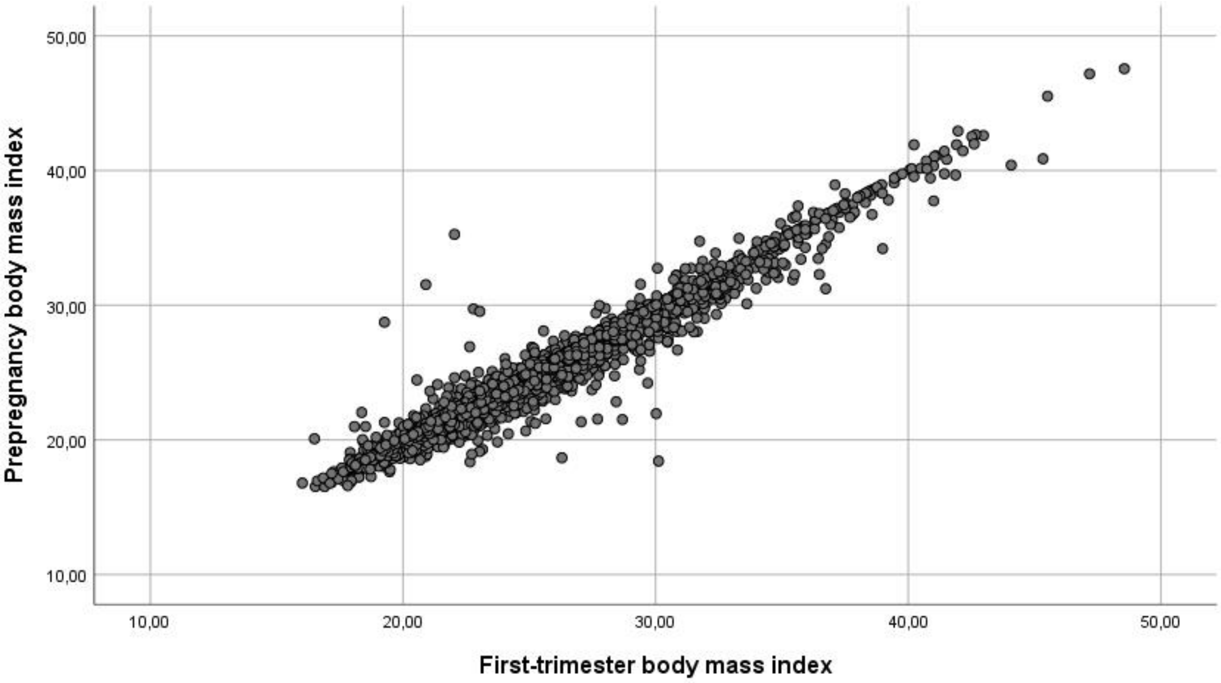

r 0.977

p-value <0.001

Imputation 4

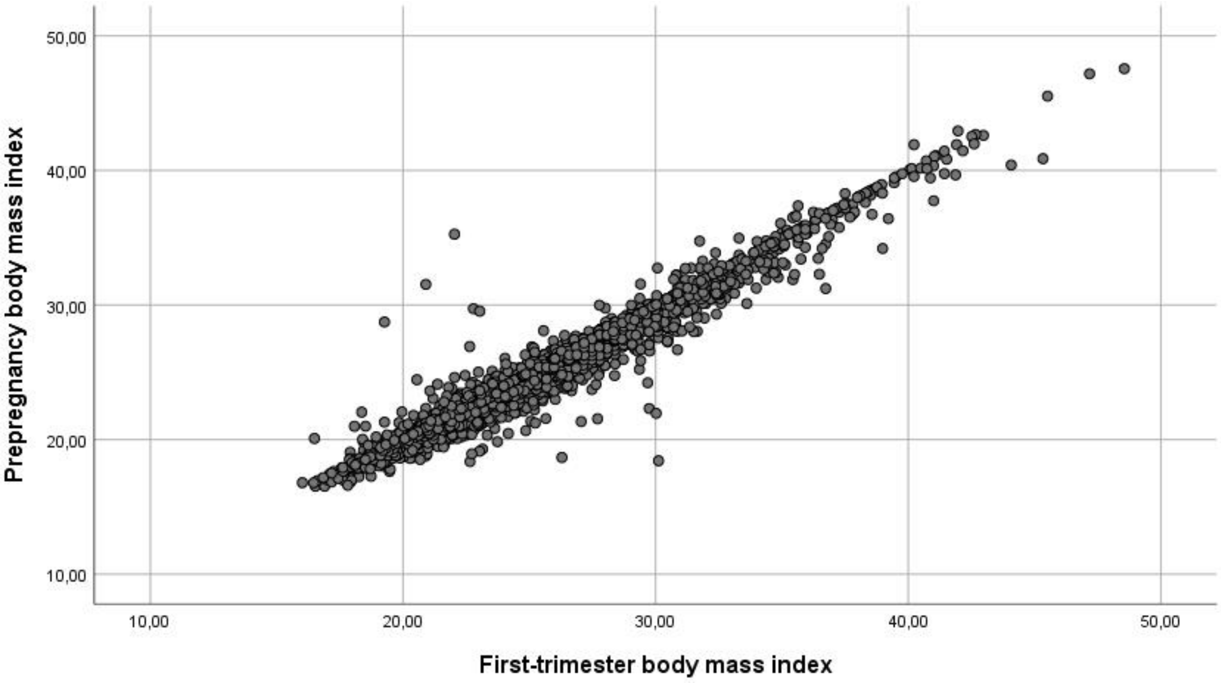

r 0.978

p-value <0.001

Imputation 5

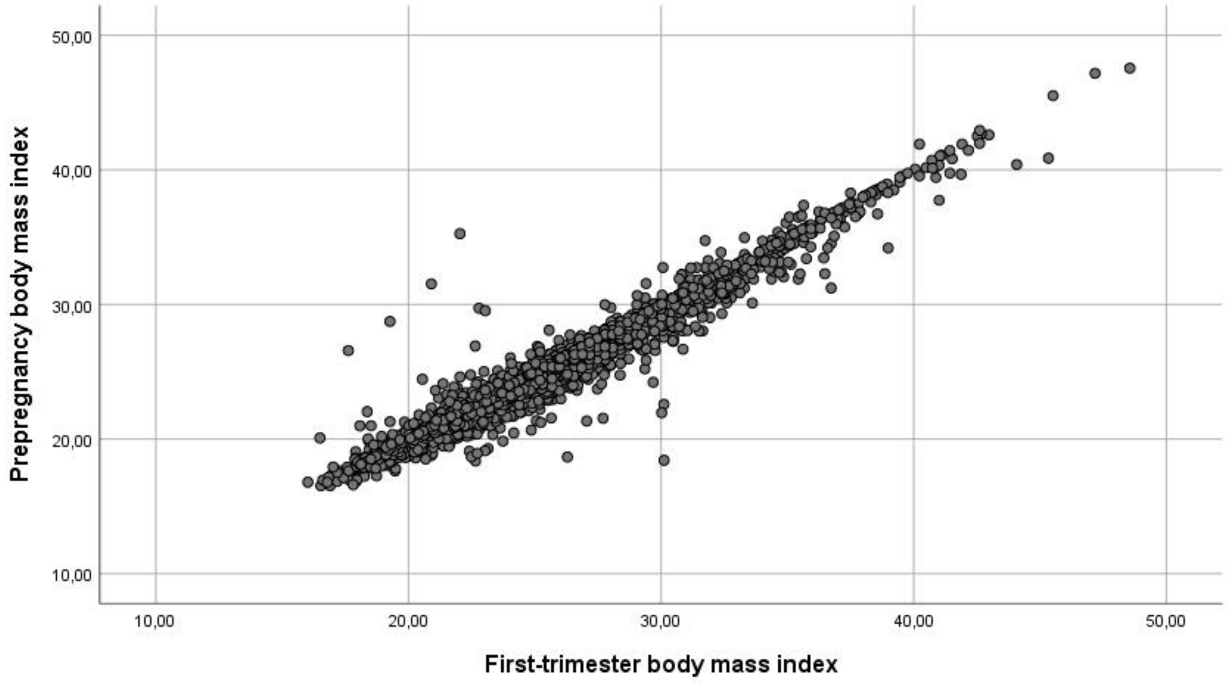

r 0.977

p-value <0.001

Imputation 6

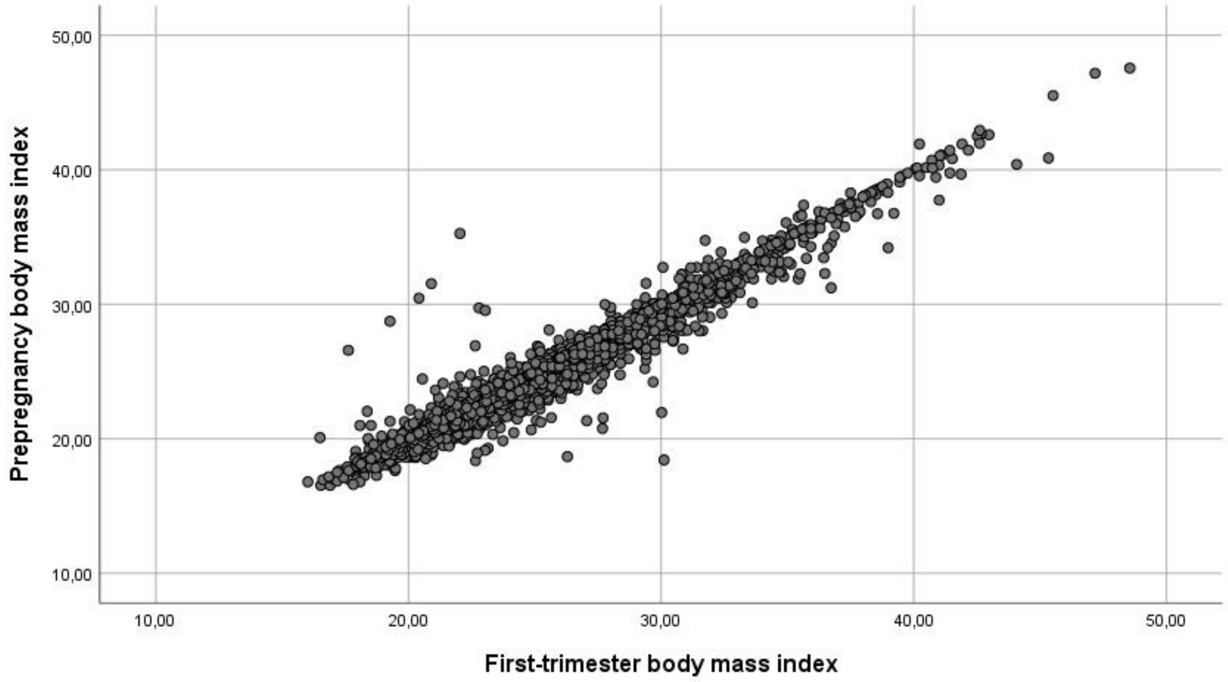

r 0.976

p-value <0.001

Imputation 7

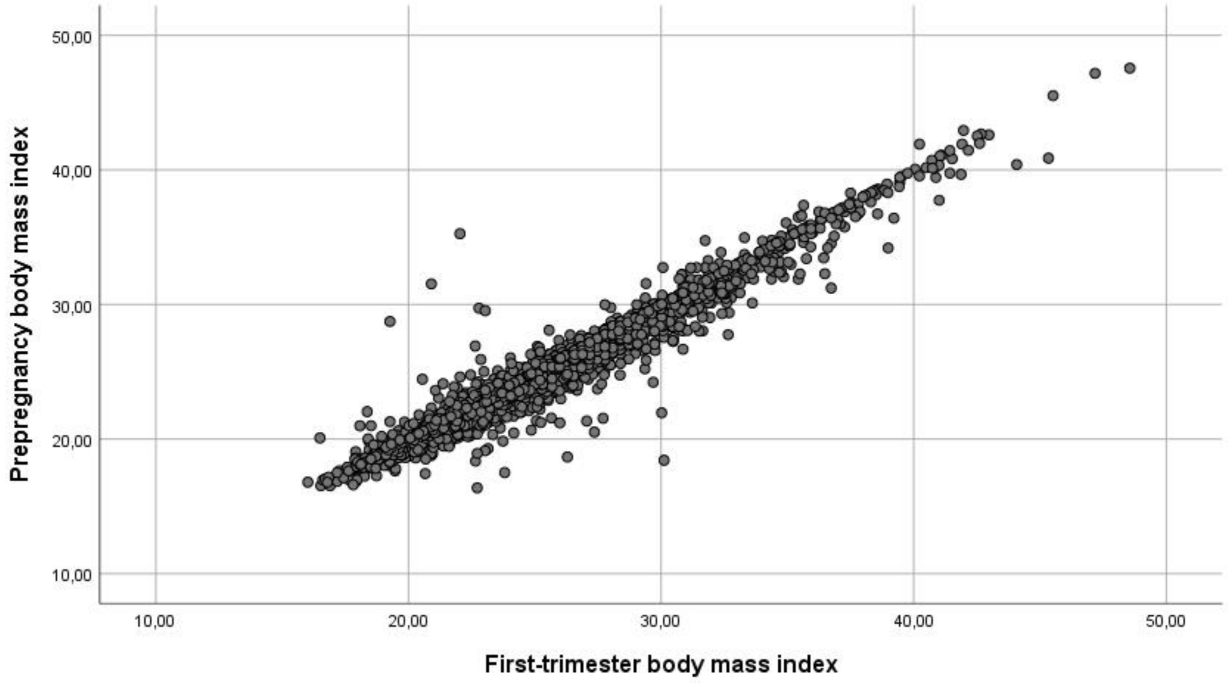

r 0.977

p-value <0.001

Imputation 8

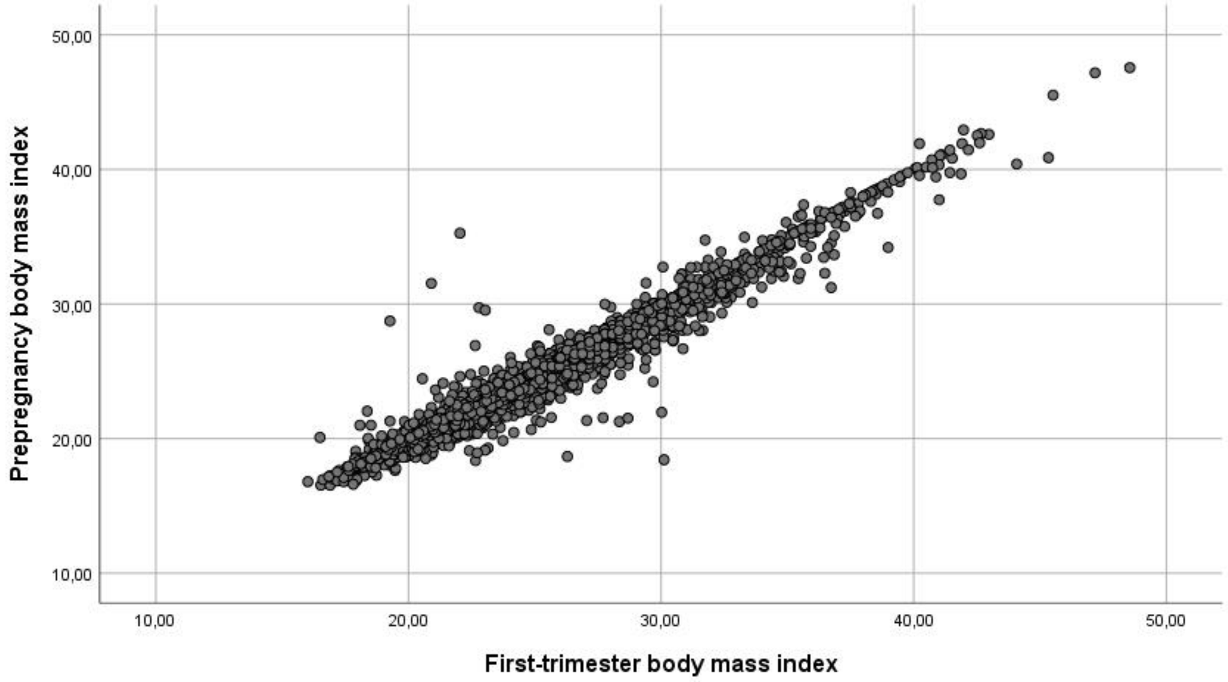

r 0.977

p-value <0.001

Imputation 9

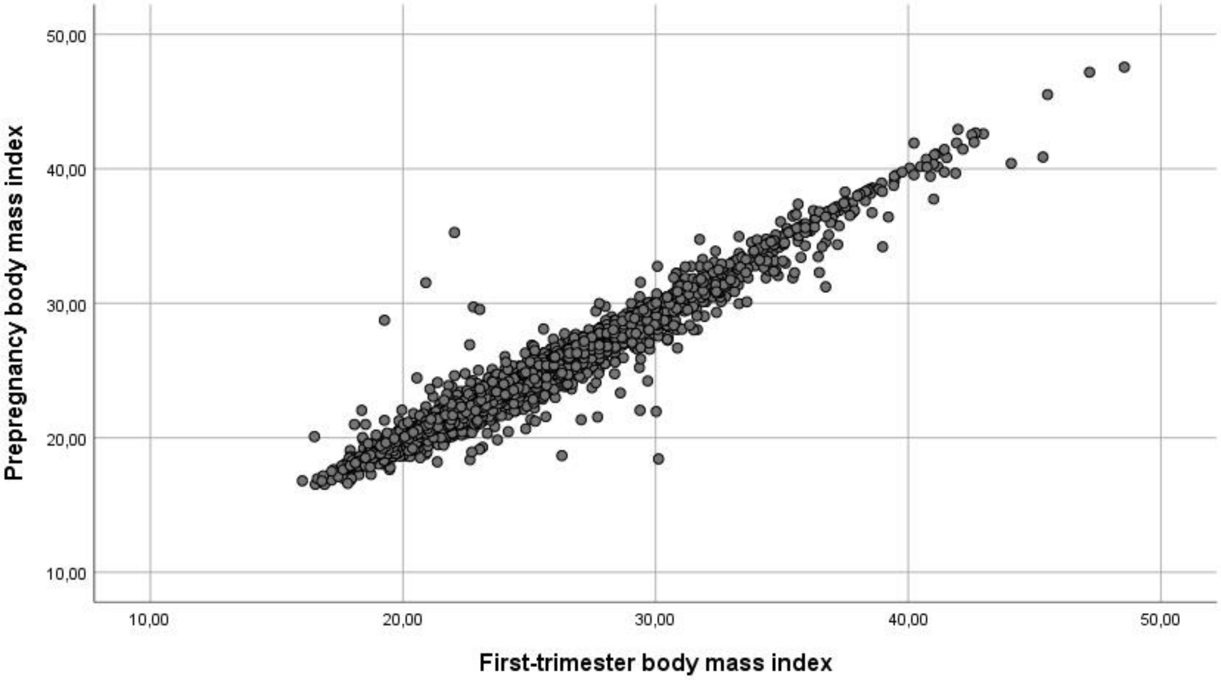

r 0.977

p-value <0.001

Imputation 10

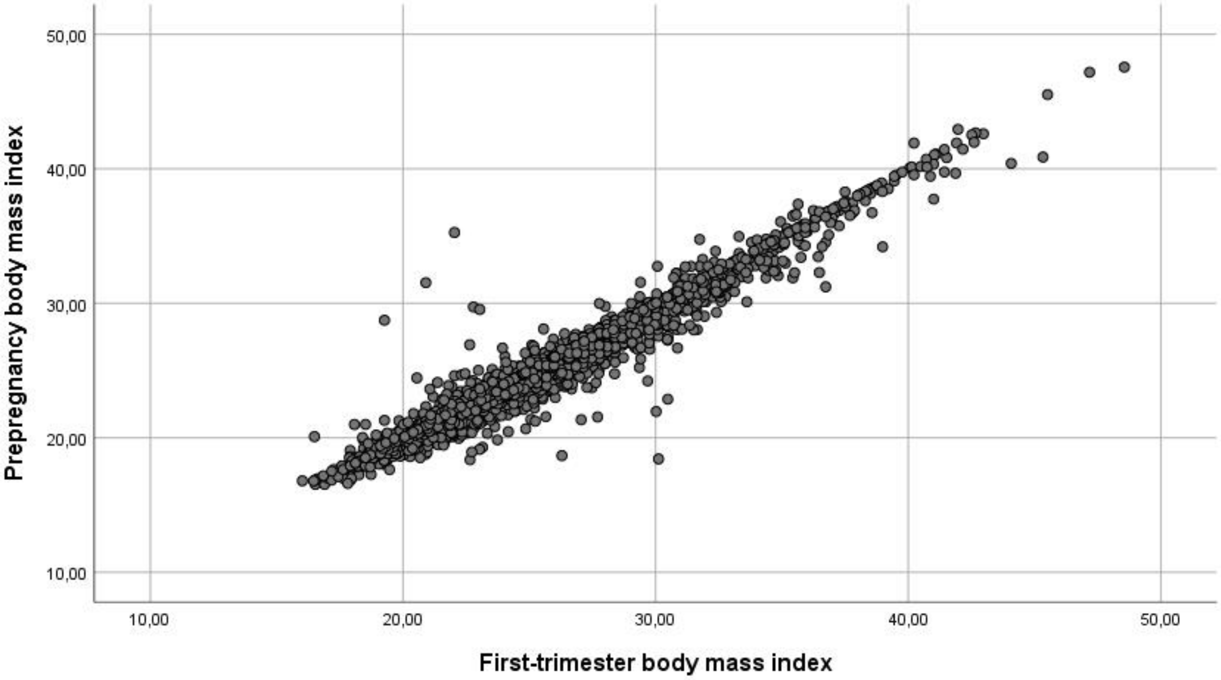

r 0.978

p-value <0.001
